# Bis[μ_2_-1,1-(butane-1,4-di­yl)-2,3-dicyclo­hexyl­guanidinato]bis­[(tetra­hydro­furan)­lithium](*Li—Li*)

**DOI:** 10.1107/S1600536810046477

**Published:** 2010-11-17

**Authors:** Hongfei Han, Wenjuan Li, Haoyang Li

**Affiliations:** a Department of Chemistry, Taiyuan Normal University, Taiyuan 030031, People’s Republic of China

## Abstract

In the dinuclear centrosymmetric title complex, [Li_2_(C_17_H_30_N_3_)_2_(C_4_H_8_O)_2_], the Li^+^ cation is coordinated by three N atoms from two guanidinate ligands and an O atom from tetra­hydro­furan (THF) in a strongly distorted tetrahedral environment. In the guanidinate-bridged THF-stabilized dimer the Li⋯Li separation is short at 2.479 (8) Å.

## Related literature

For related guanidinato compounds, see: Chandra *et al.* (1970 ▶); Barker & Kilner (1994 ▶); Bailey & Pace (2001 ▶); Coles & Hitchcock (2004 ▶); Corey *et al.* (2006 ▶); Zhou *et al.* (2007 ▶). 
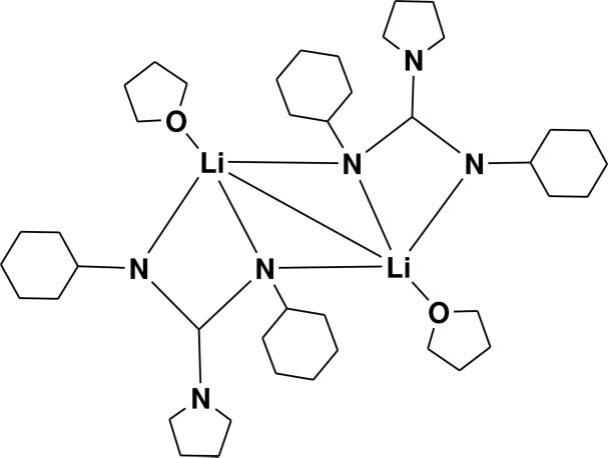


## Experimental

### 

#### Crystal data

[Li_2_(C_17_H_30_N_3_)_2_(C_4_H_8_O)_2_]
*M* *_r_* = 710.97
Monoclinic, 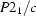

*a* = 10.446 (6) Å
*b* = 21.454 (15) Å
*c* = 10.491 (6) Å
β = 114.13 (4)°
*V* = 2146 (2) Å^3^
*Z* = 2
Mo *K*α radiation
μ = 0.07 mm^−1^
*T* = 223 K
0.30 × 0.20 × 0.20 mm

#### Data collection

Bruker SMART CCD area-detector diffractometer
Absorption correction: multi-scan (*SADABS*; Sheldrick, 1996 ▶) *T*_min_ = 0.980, *T*_max_ = 0.987
8457 measured reflections
3688 independent reflections
2749 reflections with *I* > 2σ(*I*)
*R*_int_ = 0.042

#### Refinement

*R*[*F* ^2^ > 2σ(*F* ^2^)] = 0.082
*wR*(*F* ^2^) = 0.237
*S* = 1.05
3688 reflections
235 parameters
H-atom parameters constrained
Δρ_max_ = 0.68 e Å^−3^
Δρ_min_ = −0.61 e Å^−3^

### 

Data collection: *SMART* (Bruker, 2000 ▶); cell refinement: *SAINT* (Bruker, 2000 ▶); data reduction: *SAINT*; program(s) used to solve structure: *SHELXS97* (Sheldrick, 2008 ▶); program(s) used to refine structure: *SHELXL97* (Sheldrick, 2008 ▶); molecular graphics: *SHELXTL* (Sheldrick, 2008 ▶); software used to prepare material for publication: *SHELXTL*.

## Supplementary Material

Crystal structure: contains datablocks I, global. DOI: 10.1107/S1600536810046477/jj2061sup1.cif

Structure factors: contains datablocks I. DOI: 10.1107/S1600536810046477/jj2061Isup2.hkl

Additional supplementary materials:  crystallographic information; 3D view; checkCIF report

## Figures and Tables

**Table 1:** Selected bond lengths (Å)

Li—O	1.973 (5)
Li—N2^i^	1.997 (5)
Li—N1	2.057 (5)
Li—N1^i^	2.204 (5)

Symmetry code: (i) 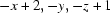.

**Fig. 1.**
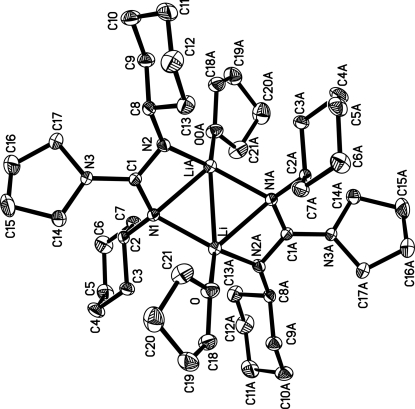
The molecular structure, showing the atom–numbering scheme. Displacement ellipsoids are drawn at the 30% probability level. H atoms have been omitted for clarity.

## supplementary crystallographic information

### Comment

As a result of the donor ability of the nitrogen centers and the potential to exploit both the steric and electronic effects induced by the programmed variation of organic substituents,the guanidinate anion has generated significant interest as a ligand (Bailey & Pace, 2001; Barker & Kilner, 1994).  Since the first guanidinato complexes have been reported (Chandra *et al.*, 1970), guanidinato ligands have been used extensively in the coordination chemistry of transition, f-block, and main-group metals (Corey *et al.*, 2006). Moreover many guanidinato complexes were reported showing good performance in ethylene polymerization (Zhou *et al.*, 2007) and in ring-opening polymerisation reactions (Coles & Hitchcock, 2004) in catalysis applications.

In the title complex,[(THF)LiN(C_6_H_11_)C(NC_4_H_8_)N(C_6_H_11_)]_2_ , the Li cation is coordinated by three N atoms from two guanidinato ligands and an O atom from tetrahydrofuran as a dimer around a planar Li/N1/LiA/N1A ring (Fig. 1).  The core of the centrosymmetric molecule has a fused tricyclic ladder motif comprising a central planar Li/N1/LiA/N1A ring flanked by planar N2/C1/N1/LiA and N2A/C1A/N1A/Li rings.  The dihedral angle between the latter two rings is 46.4 (9)°.  Inside the guanidinato-bridged THF-stabilized dimer the Li···Li separation is short at 2.479 (8) Å. Electronic delocalization throughout the guanidinate moiety is observed as evidenced by the C-N distances [N1—C1: 1.355 (3) Å, N2—C1: 1.314 (3) Å].

### Experimental

A solution of N-tetrahydropyrrolyl lithium in diethylether (0.232g, 3mmol) was added dropwise with stirring at 0 C to a solution of *N,N*'-dicyclohexyl carbodiimide (0.619g, 3mmol) in ether. The mixture was warmed to room temperature and stirred for 2h. The solvent was removed under reduced pressure. The resulting white precipitate was washed with hexane and dried *in vacuo*. The residue was dissolved in a mixed solvent of THF and hexane, and then filtered.  The concentration of the filtrate under reduced pressure gave the colorless crystals suitable for X-ray analysis over several days (yield 0.534g, 50%).

### Refinement

All of the H atoms were contrained to ideal geometry and refined under the riding model with C–H distances of 0.98-0.99 Å and U_iso_(H) = 1.2U_eq_(C).

### Crystal data

**Table:**

[Li_2_(C_17_H_30_N_3_)_2_(C_4_H_8_O)_2_]	*F*(000) = 784
*M**_r_* = 710.97	*D*_x_ = 1.100 Mg m^−^^3^
Monoclinic, *P*2_1_/*c*	Mo *K*α radiation, λ = 0.71073 Å
Hall symbol: -P 2ybc	Cell parameters from 1854 reflections
*a* = 10.446 (6) Å	θ = 1.9–25.0°
*b* = 21.454 (15) Å	µ = 0.07 mm^−^^1^
*c* = 10.491 (6) Å	*T* = 223 K
β = 114.13 (4)°	Block, colorless
*V* = 2146 (2) Å^3^	0.30 × 0.20 × 0.20 mm
*Z* = 2	

### Data collection

**Table:**

Bruker SMART CCD area-detector diffractometer	3688 independent reflections
Radiation source: fine-focus sealed tube	2749 reflections with *I* > 2σ(*I*)
graphite	*R*_int_ = 0.042
φ and ω scans	θ_max_ = 25.0°, θ_min_ = 1.9°
Absorption correction: multi-scan (*SADABS*; Sheldrick, 1996)	*h* = −12→12
*T*_min_ = 0.980, *T*_max_ = 0.987	*k* = −25→25
8457 measured reflections	*l* = −12→10

### Refinement

**Table:**

Refinement on *F*^2^	Primary atom site location: structure-invariant direct methods
Least-squares matrix: full	Secondary atom site location: difference Fourier map
*R*[*F*^2^ > 2σ(*F*^2^)] = 0.082	Hydrogen site location: inferred from neighbouring sites
*wR*(*F*^2^) = 0.237	H-atom parameters constrained
*S* = 1.05	*w* = 1/[σ^2^(*F*_o_^2^) + (0.1491*P*)^2^ + 0.6111*P*] where *P* = (*F*_o_^2^ + 2*F*_c_^2^)/3
3688 reflections	(Δ/σ)_max_ < 0.001
235 parameters	Δρ_max_ = 0.68 e Å^−^^3^
0 restraints	Δρ_min_ = −0.61 e Å^−^^3^

### Special details

**Table:**

Geometry. All esds (except the esd in the dihedral angle between two l.s. planes) are estimated using the full covariance matrix. The cell esds are taken into account individually in the estimation of esds in distances, angles and torsion angles; correlations between esds in cell parameters are only used when they are defined by crystal symmetry. An approximate (isotropic) treatment of cell esds is used for estimating esds involving l.s. planes.
Refinement. Refinement of *F*^2^ against ALL reflections. The weighted *R*-factor *wR* and goodness of fit *S* are based on *F*^2^, conventional *R*-factors *R* are based on *F*, with *F* set to zero for negative *F*^2^. The threshold expression of *F*^2^ > σ(*F*^2^) is used only for calculating *R*-factors(gt) *etc*. and is not relevant to the choice of reflections for refinement. *R*-factors based on *F*^2^ are statistically about twice as large as those based on *F*, and *R*- factors based on ALL data will be even larger.

### Fractional atomic coordinates and isotropic or equivalent isotropic displacement parameters (Å^2^)

**Table:**

	*x*	*y*	*z*	*U*_iso_*/*U*_eq_	
Li	1.0428 (4)	0.04287 (19)	0.4484 (4)	0.0287 (9)	
N1	0.8388 (2)	0.03737 (9)	0.4284 (2)	0.0258 (5)	
N2	0.7869 (2)	−0.06576 (9)	0.3820 (2)	0.0282 (5)	
N3	0.6042 (2)	0.00733 (10)	0.2715 (2)	0.0311 (6)	
O	1.0417 (2)	0.06534 (9)	0.26561 (19)	0.0396 (5)	
C1	0.7440 (2)	−0.00756 (11)	0.3611 (2)	0.0245 (6)	
C2	0.7853 (2)	0.09453 (10)	0.4653 (3)	0.0254 (6)	
H2	0.6822	0.0952	0.4121	0.030*	
C3	0.8457 (3)	0.15283 (11)	0.4260 (3)	0.0316 (6)	
H3A	0.9480	0.1527	0.4764	0.038*	
H3B	0.8232	0.1521	0.3257	0.038*	
C4	0.7868 (3)	0.21241 (13)	0.4613 (3)	0.0442 (8)	
H4A	0.8314	0.2486	0.4396	0.053*	
H4B	0.6858	0.2148	0.4032	0.053*	
C5	0.8114 (3)	0.21468 (13)	0.6146 (3)	0.0465 (8)	
H5A	0.9120	0.2188	0.6725	0.056*	
H5B	0.7639	0.2512	0.6311	0.056*	
C6	0.7565 (4)	0.15618 (14)	0.6565 (3)	0.0476 (8)	
H6A	0.6539	0.1553	0.6083	0.057*	
H6B	0.7811	0.1573	0.7572	0.057*	
C7	0.8164 (3)	0.09684 (12)	0.6211 (3)	0.0359 (7)	
H7A	0.7751	0.0603	0.6459	0.043*	
H7B	0.9180	0.0955	0.6761	0.043*	
C8	0.7020 (2)	−0.11505 (11)	0.2911 (3)	0.0284 (6)	
H8	0.6187	−0.0960	0.2164	0.034*	
C9	0.6532 (3)	−0.16101 (12)	0.3720 (3)	0.0369 (7)	
H9A	0.5972	−0.1388	0.4131	0.044*	
H9B	0.7352	−0.1787	0.4485	0.044*	
C10	0.5658 (3)	−0.21360 (14)	0.2799 (3)	0.0456 (8)	
H10A	0.5389	−0.2428	0.3366	0.055*	
H10B	0.4799	−0.1964	0.2077	0.055*	
C11	0.6480 (4)	−0.24799 (14)	0.2117 (4)	0.0545 (9)	
H11A	0.7279	−0.2693	0.2837	0.065*	
H11B	0.5879	−0.2797	0.1479	0.065*	
C12	0.7002 (4)	−0.20397 (15)	0.1320 (4)	0.0542 (9)	
H12A	0.6200	−0.1869	0.0525	0.065*	
H12B	0.7588	−0.2270	0.0952	0.065*	
C13	0.7857 (3)	−0.15020 (13)	0.2236 (3)	0.0415 (7)	
H13A	0.8125	−0.1213	0.1665	0.050*	
H13B	0.8718	−0.1668	0.2968	0.050*	
C14	0.5631 (3)	0.05740 (13)	0.1696 (3)	0.0355 (7)	
H14A	0.5720	0.0446	0.0839	0.043*	
H14B	0.6209	0.0945	0.2072	0.043*	
C15	0.4109 (4)	0.0700 (2)	0.1417 (5)	0.0731 (12)	
H15A	0.3945	0.1149	0.1436	0.088*	
H15B	0.3486	0.0536	0.0499	0.088*	
C16	0.3846 (4)	0.0377 (2)	0.2543 (5)	0.0751 (13)	
H16A	0.4007	0.0661	0.3326	0.090*	
H16B	0.2879	0.0224	0.2191	0.090*	
C17	0.4850 (3)	−0.01453 (14)	0.2982 (3)	0.0431 (7)	
H17A	0.4439	−0.0521	0.2436	0.052*	
H17B	0.5136	−0.0238	0.3976	0.052*	
C18	1.0836 (3)	0.12480 (14)	0.2329 (3)	0.0439 (8)	
H18A	1.1860	0.1283	0.2730	0.053*	
H18B	1.0456	0.1586	0.2699	0.053*	
C19	1.0247 (4)	0.12776 (17)	0.0757 (4)	0.0571 (9)	
H19A	1.0054	0.1708	0.0421	0.069*	
H19B	1.0884	0.1085	0.0398	0.069*	
C20	0.8910 (4)	0.09066 (19)	0.0352 (4)	0.0636 (10)	
H20A	0.8603	0.0738	−0.0595	0.076*	
H20B	0.8156	0.1162	0.0406	0.076*	
C21	0.9332 (3)	0.03918 (16)	0.1427 (3)	0.0501 (8)	
H21A	0.8531	0.0260	0.1621	0.060*	
H21B	0.9685	0.0030	0.1097	0.060*	

### Atomic displacement parameters (Å^2^)

**Table:**

	*U*^11^	*U*^22^	*U*^33^	*U*^12^	*U*^13^	*U*^23^
Li	0.023 (2)	0.028 (2)	0.032 (2)	−0.0009 (17)	0.0092 (18)	0.0017 (17)
N1	0.0210 (11)	0.0210 (10)	0.0326 (12)	−0.0009 (8)	0.0081 (9)	−0.0012 (8)
N2	0.0259 (11)	0.0206 (11)	0.0306 (12)	0.0000 (8)	0.0039 (9)	−0.0021 (8)
N3	0.0183 (11)	0.0282 (11)	0.0417 (13)	−0.0008 (8)	0.0070 (9)	0.0063 (9)
O	0.0414 (12)	0.0434 (12)	0.0326 (11)	−0.0087 (9)	0.0137 (9)	0.0035 (8)
C1	0.0214 (13)	0.0262 (13)	0.0261 (13)	−0.0007 (10)	0.0099 (10)	0.0014 (10)
C2	0.0208 (12)	0.0216 (12)	0.0316 (14)	0.0022 (9)	0.0085 (10)	0.0003 (10)
C3	0.0350 (15)	0.0244 (13)	0.0347 (15)	−0.0012 (11)	0.0134 (12)	0.0015 (10)
C4	0.0527 (19)	0.0236 (14)	0.0531 (19)	0.0045 (12)	0.0185 (15)	0.0043 (13)
C5	0.0525 (19)	0.0311 (15)	0.0538 (19)	0.0050 (13)	0.0197 (15)	−0.0099 (13)
C6	0.062 (2)	0.0454 (18)	0.0457 (18)	0.0076 (15)	0.0319 (16)	−0.0051 (14)
C7	0.0409 (16)	0.0305 (14)	0.0397 (16)	0.0016 (12)	0.0201 (13)	0.0028 (12)
C8	0.0235 (13)	0.0246 (13)	0.0307 (14)	−0.0004 (10)	0.0044 (11)	−0.0011 (10)
C9	0.0390 (16)	0.0295 (14)	0.0390 (16)	−0.0051 (12)	0.0128 (13)	−0.0024 (12)
C10	0.0472 (18)	0.0338 (16)	0.0488 (18)	−0.0121 (13)	0.0124 (14)	−0.0023 (13)
C11	0.054 (2)	0.0286 (16)	0.065 (2)	−0.0043 (14)	0.0080 (17)	−0.0133 (15)
C12	0.056 (2)	0.0511 (19)	0.057 (2)	−0.0076 (16)	0.0242 (17)	−0.0267 (16)
C13	0.0385 (16)	0.0404 (16)	0.0456 (18)	−0.0043 (13)	0.0173 (14)	−0.0121 (13)
C14	0.0298 (15)	0.0366 (15)	0.0327 (15)	0.0005 (11)	0.0052 (12)	0.0041 (12)
C15	0.042 (2)	0.083 (3)	0.088 (3)	0.0260 (19)	0.019 (2)	0.043 (2)
C16	0.042 (2)	0.067 (2)	0.123 (4)	0.0193 (18)	0.041 (2)	0.034 (2)
C17	0.0260 (15)	0.0427 (17)	0.060 (2)	0.0015 (12)	0.0165 (14)	0.0078 (14)
C18	0.0455 (18)	0.0397 (16)	0.0545 (19)	−0.0028 (13)	0.0285 (15)	0.0038 (14)
C19	0.063 (2)	0.060 (2)	0.054 (2)	0.0105 (17)	0.0285 (18)	0.0237 (17)
C20	0.055 (2)	0.085 (3)	0.043 (2)	0.0093 (19)	0.0131 (17)	0.0083 (18)
C21	0.052 (2)	0.063 (2)	0.0344 (16)	−0.0132 (16)	0.0162 (15)	−0.0061 (15)

### Geometric parameters (Å, °)

**Table:**

Li—O	1.973 (5)	C8—H8	0.9900
Li—N2^i^	1.997 (5)	C9—C10	1.522 (4)
Li—N1	2.057 (5)	C9—H9A	0.9800
Li—N1^i^	2.204 (5)	C9—H9B	0.9800
Li—C1^i^	2.427 (5)	C10—C11	1.515 (5)
Li—Li^i^	2.479 (8)	C10—H10A	0.9800
N1—C1	1.355 (3)	C10—H10B	0.9800
N1—C2	1.464 (3)	C11—C12	1.504 (5)
N1—Li^i^	2.204 (5)	C11—H11A	0.9800
N2—C1	1.314 (3)	C11—H11B	0.9800
N2—C8	1.457 (3)	C12—C13	1.533 (4)
N2—Li^i^	1.997 (5)	C12—H12A	0.9800
N3—C1	1.413 (3)	C12—H12B	0.9800
N3—C14	1.451 (3)	C13—H13A	0.9800
N3—C17	1.461 (4)	C13—H13B	0.9800
O—C18	1.435 (4)	C14—C15	1.518 (4)
O—C21	1.438 (4)	C14—H14A	0.9800
C1—Li^i^	2.427 (5)	C14—H14B	0.9800
C2—C7	1.532 (4)	C15—C16	1.489 (6)
C2—C3	1.531 (3)	C15—H15A	0.9800
C2—H2	0.9900	C15—H15B	0.9800
C3—C4	1.528 (4)	C16—C17	1.475 (5)
C3—H3A	0.9800	C16—H16A	0.9800
C3—H3B	0.9800	C16—H16B	0.9800
C4—C5	1.523 (5)	C17—H17A	0.9800
C4—H4A	0.9800	C17—H17B	0.9800
C4—H4B	0.9800	C18—C19	1.507 (5)
C5—C6	1.518 (4)	C18—H18A	0.9800
C5—H5A	0.9800	C18—H18B	0.9800
C5—H5B	0.9800	C19—C20	1.510 (5)
C6—C7	1.530 (4)	C19—H19A	0.9800
C6—H6A	0.9800	C19—H19B	0.9800
C6—H6B	0.9800	C20—C21	1.510 (5)
C7—H7A	0.9800	C20—H20A	0.9800
C7—H7B	0.9800	C20—H20B	0.9800
C8—C9	1.519 (4)	C21—H21A	0.9800
C8—C13	1.529 (4)	C21—H21B	0.9800
			
O—Li—N2^i^	117.1 (2)	C8—C9—C10	112.2 (2)
O—Li—N1	108.7 (2)	C8—C9—H9A	109.2
N2^i^—Li—N1	127.9 (2)	C10—C9—H9A	109.2
O—Li—N1^i^	122.7 (2)	C8—C9—H9B	109.2
N2^i^—Li—N1^i^	65.61 (15)	C10—C9—H9B	109.2
N1—Li—N1^i^	108.94 (18)	H9A—C9—H9B	107.9
O—Li—C1^i^	121.0 (2)	C11—C10—C9	110.6 (3)
N2^i^—Li—C1^i^	32.77 (10)	C11—C10—H10A	109.5
N1—Li—C1^i^	129.0 (2)	C9—C10—H10A	109.5
N1^i^—Li—C1^i^	33.59 (10)	C11—C10—H10B	109.5
O—Li—Li^i^	138.3 (3)	C9—C10—H10B	109.5
N2^i^—Li—Li^i^	98.2 (2)	H10A—C10—H10B	108.1
N1—Li—Li^i^	57.24 (16)	C12—C11—C10	111.2 (3)
N1^i^—Li—Li^i^	51.70 (16)	C12—C11—H11A	109.4
C1^i^—Li—Li^i^	77.4 (2)	C10—C11—H11A	109.4
C1—N1—C2	117.2 (2)	C12—C11—H11B	109.4
C1—N1—Li	126.8 (2)	C10—C11—H11B	109.4
C2—N1—Li	114.63 (19)	H11A—C11—H11B	108.0
C1—N1—Li^i^	82.27 (18)	C11—C12—C13	112.0 (3)
C2—N1—Li^i^	133.03 (19)	C11—C12—H12A	109.2
Li—N1—Li^i^	71.06 (18)	C13—C12—H12A	109.2
C1—N2—C8	120.4 (2)	C11—C12—H12B	109.2
C1—N2—Li^i^	91.93 (19)	C13—C12—H12B	109.2
C8—N2—Li^i^	147.6 (2)	H12A—C12—H12B	107.9
C1—N3—C14	124.9 (2)	C12—C13—C8	111.4 (2)
C1—N3—C17	122.0 (2)	C12—C13—H13A	109.3
C14—N3—C17	111.1 (2)	C8—C13—H13A	109.3
C18—O—C21	109.8 (2)	C12—C13—H13B	109.3
C18—O—Li	124.6 (2)	C8—C13—H13B	109.3
C21—O—Li	117.7 (2)	H13A—C13—H13B	108.0
N2—C1—N1	117.6 (2)	N3—C14—C15	104.2 (2)
N2—C1—N3	121.0 (2)	N3—C14—H14A	110.9
N1—C1—N3	121.5 (2)	C15—C14—H14A	110.9
N2—C1—Li^i^	55.31 (16)	N3—C14—H14B	110.9
N1—C1—Li^i^	64.14 (16)	C15—C14—H14B	110.9
N3—C1—Li^i^	165.82 (19)	H14A—C14—H14B	108.9
N1—C2—C7	111.85 (19)	C16—C15—C14	106.5 (3)
N1—C2—C3	111.65 (19)	C16—C15—H15A	110.4
C7—C2—C3	109.3 (2)	C14—C15—H15A	110.4
N1—C2—H2	108.0	C16—C15—H15B	110.4
C7—C2—H2	108.0	C14—C15—H15B	110.4
C3—C2—H2	108.0	H15A—C15—H15B	108.6
C4—C3—C2	111.5 (2)	C17—C16—C15	105.2 (3)
C4—C3—H3A	109.3	C17—C16—H16A	110.7
C2—C3—H3A	109.3	C15—C16—H16A	110.7
C4—C3—H3B	109.3	C17—C16—H16B	110.7
C2—C3—H3B	109.3	C15—C16—H16B	110.7
H3A—C3—H3B	108.0	H16A—C16—H16B	108.8
C5—C4—C3	111.8 (2)	C16—C17—N3	104.4 (3)
C5—C4—H4A	109.2	C16—C17—H17A	110.9
C3—C4—H4A	109.2	N3—C17—H17A	110.9
C5—C4—H4B	109.2	C16—C17—H17B	110.9
C3—C4—H4B	109.2	N3—C17—H17B	110.9
H4A—C4—H4B	107.9	H17A—C17—H17B	108.9
C6—C5—C4	111.0 (2)	O—C18—C19	105.7 (3)
C6—C5—H5A	109.4	O—C18—H18A	110.6
C4—C5—H5A	109.4	C19—C18—H18A	110.6
C6—C5—H5B	109.4	O—C18—H18B	110.6
C4—C5—H5B	109.4	C19—C18—H18B	110.6
H5A—C5—H5B	108.0	H18A—C18—H18B	108.7
C5—C6—C7	112.2 (2)	C20—C19—C18	101.7 (3)
C5—C6—H6A	109.2	C20—C19—H19A	111.4
C7—C6—H6A	109.2	C18—C19—H19A	111.4
C5—C6—H6B	109.2	C20—C19—H19B	111.4
C7—C6—H6B	109.2	C18—C19—H19B	111.4
H6A—C6—H6B	107.9	H19A—C19—H19B	109.3
C6—C7—C2	110.9 (2)	C19—C20—C21	102.8 (3)
C6—C7—H7A	109.5	C19—C20—H20A	111.2
C2—C7—H7A	109.5	C21—C20—H20A	111.2
C6—C7—H7B	109.5	C19—C20—H20B	111.2
C2—C7—H7B	109.5	C21—C20—H20B	111.2
H7A—C7—H7B	108.0	H20A—C20—H20B	109.1
N2—C8—C9	111.0 (2)	O—C21—C20	105.5 (3)
N2—C8—C13	110.6 (2)	O—C21—H21A	110.6
C9—C8—C13	109.0 (2)	C20—C21—H21A	110.6
N2—C8—H8	108.8	O—C21—H21B	110.6
C9—C8—H8	108.8	C20—C21—H21B	110.6
C13—C8—H8	108.8	H21A—C21—H21B	108.8
			
O—Li—N1—C1	71.7 (3)	C17—N3—C1—Li^i^	−9.3 (9)
N2^i^—Li—N1—C1	−137.6 (3)	C1—N1—C2—C7	104.0 (2)
N1^i^—Li—N1—C1	−64.2 (3)	Li—N1—C2—C7	−88.3 (2)
C1^i^—Li—N1—C1	−95.4 (3)	Li^i^—N1—C2—C7	−1.8 (3)
Li^i^—Li—N1—C1	−64.2 (3)	C1—N1—C2—C3	−133.2 (2)
O—Li—N1—C2	−94.5 (2)	Li—N1—C2—C3	34.5 (3)
N2^i^—Li—N1—C2	56.1 (3)	Li^i^—N1—C2—C3	121.0 (2)
N1^i^—Li—N1—C2	129.5 (2)	N1—C2—C3—C4	178.6 (2)
C1^i^—Li—N1—C2	98.3 (3)	C7—C2—C3—C4	−57.2 (3)
Li^i^—Li—N1—C2	129.5 (2)	C2—C3—C4—C5	55.9 (3)
O—Li—N1—Li^i^	136.0 (3)	C3—C4—C5—C6	−53.3 (4)
N2^i^—Li—N1—Li^i^	−73.4 (3)	C4—C5—C6—C7	53.9 (4)
N1^i^—Li—N1—Li^i^	0.0	C5—C6—C7—C2	−56.6 (3)
C1^i^—Li—N1—Li^i^	−31.19 (16)	N1—C2—C7—C6	−178.7 (2)
N2^i^—Li—O—C18	−45.7 (3)	C3—C2—C7—C6	57.2 (3)
N1—Li—O—C18	108.6 (3)	C1—N2—C8—C9	−116.5 (3)
N1^i^—Li—O—C18	−122.8 (3)	Li^i^—N2—C8—C9	58.7 (4)
C1^i^—Li—O—C18	−83.0 (3)	C1—N2—C8—C13	122.5 (3)
Li^i^—Li—O—C18	170.0 (4)	Li^i^—N2—C8—C13	−62.4 (4)
N2^i^—Li—O—C21	168.5 (2)	N2—C8—C9—C10	−179.2 (2)
N1—Li—O—C21	−37.3 (3)	C13—C8—C9—C10	−57.2 (3)
N1^i^—Li—O—C21	91.4 (3)	C8—C9—C10—C11	57.5 (3)
C1^i^—Li—O—C21	131.1 (3)	C9—C10—C11—C12	−55.1 (3)
Li^i^—Li—O—C21	24.1 (5)	C10—C11—C12—C13	54.6 (4)
C8—N2—C1—N1	−166.2 (2)	C11—C12—C13—C8	−55.4 (4)
Li^i^—N2—C1—N1	16.4 (2)	N2—C8—C13—C12	177.7 (2)
C8—N2—C1—N3	13.7 (3)	C9—C8—C13—C12	55.5 (3)
Li^i^—N2—C1—N3	−163.7 (2)	C1—N3—C14—C15	−159.4 (3)
C8—N2—C1—Li^i^	177.4 (3)	C17—N3—C14—C15	4.6 (3)
C2—N1—C1—N2	−149.7 (2)	N3—C14—C15—C16	13.9 (4)
Li—N1—C1—N2	44.3 (3)	C14—C15—C16—C17	−27.0 (5)
Li^i^—N1—C1—N2	−14.9 (2)	C15—C16—C17—N3	29.2 (4)
C2—N1—C1—N3	30.4 (3)	C1—N3—C17—C16	143.2 (3)
Li—N1—C1—N3	−135.6 (2)	C14—N3—C17—C16	−21.4 (4)
Li^i^—N1—C1—N3	165.1 (2)	C21—O—C18—C19	−14.8 (3)
C2—N1—C1—Li^i^	−134.8 (2)	Li—O—C18—C19	−162.9 (2)
Li—N1—C1—Li^i^	59.3 (3)	O—C18—C19—C20	32.7 (3)
C14—N3—C1—N2	−136.5 (3)	C18—C19—C20—C21	−37.6 (3)
C17—N3—C1—N2	61.1 (3)	C18—O—C21—C20	−9.5 (3)
C14—N3—C1—N1	43.4 (4)	Li—O—C21—C20	141.2 (3)
C17—N3—C1—N1	−118.9 (3)	C19—C20—C21—O	29.7 (3)
C14—N3—C1—Li^i^	153.1 (7)		

Symmetry codes: (i) −*x*+2, −*y*, −*z*+1.
